# Mapping the landscape of metabolic goals of a cell

**DOI:** 10.1186/s13059-016-0968-2

**Published:** 2016-05-23

**Authors:** Qi Zhao, Arion I. Stettner, Ed Reznik, Ioannis Ch. Paschalidis, Daniel Segrè

**Affiliations:** Department of Electrical and Computer Engineering, and Division of Systems Engineering, Boston University, Boston, MA 02215 USA; Department of Biomedical Engineering, Boston University, Boston, MA 02215 USA; Current address: Memorial Sloan Kettering Cancer Center, New York, NY 10065 USA; Department of Biology and Bioinformatics Program, Boston University, Boston, MA 02215 USA

**Keywords:** Metabolic networks, Flux balance analysis, Inverse optimization, Objective functions, Genome-scale stoichiometric models

## Abstract

Genome-scale flux balance models of metabolism provide testable predictions of all metabolic rates in an organism, by assuming that the cell is optimizing a metabolic goal known as the objective function. We introduce an efficient inverse flux balance analysis (invFBA) approach, based on linear programming duality, to characterize the space of possible objective functions compatible with measured fluxes. After testing our algorithm on simulated *E. coli* data and time-dependent *S. oneidensis* fluxes inferred from gene expression, we apply our inverse approach to flux measurements in long-term evolved *E. coli* strains, revealing objective functions that provide insight into metabolic adaptation trajectories.

**Electronic supplementary material:**

The online version of this article (doi:10.1186/s13059-016-0968-2) contains supplementary material, which is available to authorized users.

## Background

Metabolism, the chemical network that transforms nutrients supplied by the environment into energy and molecular building blocks, is one of the few cellular subsystems for which systems biology approaches can provide quantitative, testable predictions at a genomic scale. Flux balance analysis (FBA), in particular, makes it possible to simulate reaction fluxes through a stoichiometric constraint-based model of metabolism (see more details about FBA in Additional file 1: Supplementary material). FBA relies on the assumption that the metabolism of a cell has evolved to optimize an objective function, a linear combination of reactions which, in most implementations to be found in the literature, is simply the biomass reaction [1, 2]. Unlike the enzyme-catalyzed, mass-balanced reactions that make up the bulk of metabolic networks, the biomass reaction simulates growth by converting amino acids, lipids, nucleic acids, and other molecular building blocks into a unit of biomass in fixed proportions based on experimental measurements of a cell’s chemical composition.

While maximization of the growth flux constitutes a convenient, useful, and often sufficiently accurate assumption for applications of stoichiometric modeling, one should, in general, consider it as the mathematical formulation of an evolutionary hypothesis about the criteria for natural selection in unicellular organisms. Along these lines, deviations and alternatives to the widespread adoption of the biomass reaction as the objective function have been observed and proposed. For example, throughout the years, a host of alternative objective functions have been shown to be biologically relevant, including minimization of ATP production [3], minimization of the total sum of flux intensities [4], and minimization of flux redistribution upon gene deletion relative to wild type [5]. In a recent work, metabolism in evolved strains of *E. coli* was shown to migrate away from optimal efficiency as predicted by FBA when maximizing biomass production [6]. Moreover, a couple of studies have targeted the possible effects of variable biomass composition on FBA predictions [7, 8].

In general, identifying the objective that most accurately predicts cellular metabolism under a given condition can be viewed as a way to improve FBA calculations, as well as an avenue to advance our understanding of metabolism and its evolution. By dynamically regulating transcription and translation of different enzymes, and by allosterically fine-tuning their catalytic activities, the cell can distribute flux through the thousands of reactions that make up its metabolic network in a dizzying number of ways. The question we pose is whether it is possible to use the flux balance framework to associate possible metabolic objective functions to a given measured set of genome-scale fluxes. In other words, we seek to understand whether it is possible to say that a given organism was optimized to favor some reactions at the expense of others.

Those few attempts made to date at solving the FBA inverse problem (going from fluxes back to objectives) show promising results, but also a range of serious limitations, mainly stemming from the non-convexity of the proposed formulations, which lead to computationally expensive solution approaches that fail to guarantee global optimality [8, 9]. An alternative approach to estimating a true objective function [10] uses a Bayesian framework, which relies on the assumption of normally distributed experimental fluxes and does not exploit the structure of the FBA problem. To fill the knowledge gap at the heart of FBA and dispel mere biological intuition with credible objective functions that reflect internal and external metabolic fluxes measured in the lab, a new, computationally efficient method is required. Beyond identifying a single suitable objective function, it should mathematically capture the space of all possible objectives compatible with a given set of flux measurements, even noisy ones.

Here we develop a novel framework called invFBA (inverse FBA) to rigorously infer objective functions from such sets of intracellular fluxes as can be measured for central carbon metabolism with ^13^C-labeled substrates. Our invFBA formulation, based on linear optimization, guarantees global optimality and can be solved in polynomial time, unlike [8] and [9], respectively. Moreover, the output of invFBA has a meaningful biological interpretation. We begin by stating the mathematical formulation of invFBA and the regularization procedure. We next test invFBA on simulated *E. coli* fluxes, with and without noise, in order to assess its performance. After that, we validate our approach using time-dependent fluxes inferred from gene expression data. Finally, we apply our method to fluxes measured in the central carbon metabolism of ancestral and evolved *E. coli* strains.

## Results

### InvFBA recovers known objective from simulated *E. coli* fluxes

**Fig. 1:**
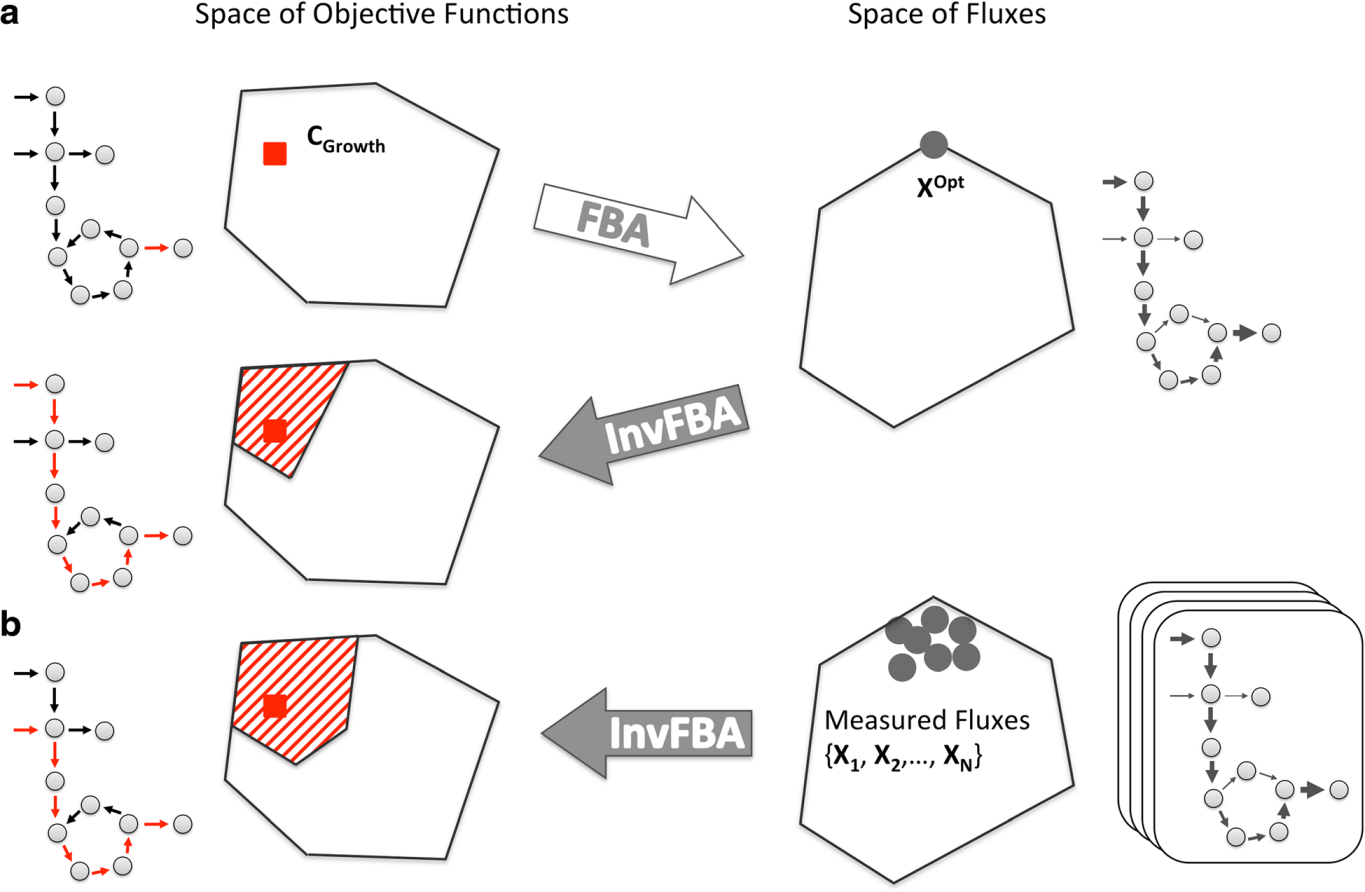
*Schematic representation* of how FBA and invFBA work. This *diagram* illustrates concisely the flow of information for invFBA calculations in this work. The *right* part of the figure displays schematic representations of the set of metabolic fluxes. Each flux vector can also be visualized on a *metabolic chart* (*right-most* part of the figure), where *gray arrows* of different thicknesses indicate different intensities of reaction fluxes throughout a network. The *left* part of the figure displays instead the space of metabolic objectives. Coefficients of the objective function can also be visualized on a *metabolic chart* (*left-most* part of the figure), with *red arrows* representing non-zero components of the objective. **a** FBA uses a given objective function (here **c**_**growth**_) to predict a set of fluxes (**X** ^**Opt**^), or multiple equivalent sets of fluxes (not shown). From one FBA solution, one can use invFBA to infer possible objective functions. The solution is not necessarily unique, though the space of possible solutions can be rigorously characterized, and contains the original objective function. **b** InvFBA can be applied to multiple (noisy) experimental measurements of fluxes, leading, as in the test case of (**a**), to a space of possible objective functions

**Fig. 2:**
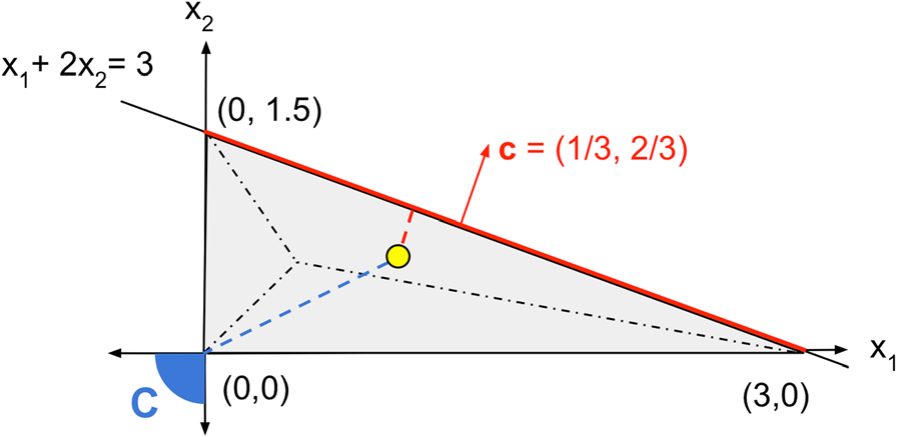
A *toy model* illustrating invFBA. To illustrate the use of the invFBA algorithm, we applied it to a simple metabolic network with a single metabolite A and three reactions, described by the stoichiometric matrix **S** = [1, 2, -1]. This corresponds essentially to two reactions (with fluxes x_1_ and x_2_) producing A, and one reaction (with flux x_3_) consuming it. We additionally impose that all fluxes are non-negative and that x_3_ ≤ 3. Thus, the feasible space is represented by the *polyhedron* {**x** | **Sx** = 0, **x** ≥ 0, x_3_ ≤ 3}, corresponding to the *triangle* in the (x_1_, x_2_) plane shown in the figure. Given a specific metabolic flux vector (*yellow dot*), we use invFBA to identify an objective function that would give such a point as an FBA optimum. In this case, invFBA yields **c** = (1/3, 2/3) as the objective. This corresponds to the *vector* perpendicular to the optimal facet closest to the given flux point. Note that the alternative possibility of seeking the extreme point of the FBA polytope closest to the *yellow dot* (as done in [4, 5]), would yield the faraway extreme point (x_1_, x_2_) = (0, 0) and an objective function within the *blue cone* C that renders this point

The objective function in FBA (Fig. 1) is encoded by a vector **c**, whose elements represent the extent to which individual fluxes tend to be maximized or minimized in the resource allocation problem that the cell tries to solve. Mathematically, the linear combination of fluxes being maximized or minimized is expressed in the form ∑_*j* = 1_^*n*^*c*_*j*_*x*_*j*_, where n is the total number of reactions in the model. The problem addressed by invFBA is to infer, from measurements of the fluxes through a cell’s metabolism under a given condition, the vector **c** that best represents its objective. Most FBA calculations include only one non-zero element in **c**, corresponding to the biomass production flux. In our invFBA approach, we want to assume that more complex **c** vectors may better capture the objective function implied by experimentally measured fluxes. The intuition behind invFBA is also described through a simple toy model in Fig. 2.

Before applying invFBA to experimental measurements of a cell’s metabolism whose underlying objective is unknown, we first tested invFBA on *in silico* fluxes simulated by FBA with a known objective. Using the iJO1366 metabolic model for *E. coli* [11], we simulated growth in a standard minimal medium (MOPS) under three different carbon source limitations: glucose, succinate, and glycerol. In all cases, the objective function was chosen so that FBA maximizes the biomass reaction flux. We next used the output flux vectors predicted by FBA (which we will refer to as the “observed fluxes”) as an input to invFBA. The invFBA algorithm tries to infer possible objective functions that could yield the observed fluxes as solutions in FBA. Our standard formulation of invFBA works in two steps: the first step identifies a set of objective functions compatible with the observed fluxes; the second step narrows down this set to a putative sparse objective, with a minimal L1 norm. A third step is alternatively used to find the sparsest objective (which has a minimal number of non-zero elements in the objective function) if needed (see details in “Methods,” and an alternative single-step LASSO formulation in Additional file 1: Supplementary Methods).

Upon applying invFBA to the FBA-generated observed fluxes, we found that the algorithm correctly recovered maximization of the biomass flux in all three conditions (inferred coefficients are shown in Additional file 2: Table S7). One immediate question is whether this solution is unique. In order to explore the spectrum of possible equivalent invFBA solutions, we extended to invFBA the method of flux variability analysis often used in classical FBA calculations [1]. In this case, we wanted to characterize the possible range for each possible element in the objective function vector **c**. This method, which we call objective variability analysis (OVA), determines the full range of values each coefficient of the objective function can assume while being consistent with optimality (see Additional file 1: Supplementary Methods for details). By running OVA on these test cases, we found that while invFBA yielded maximization of biomass as a solution under all conditions, alternative objective functions were equally compatible with the observed fluxes under the different conditions (Additional file 3: Table S1, Additional file 4: Table S2, and Additional file 5: Table S3). For instance, under succinate-limited conditions, an equivalent objective function is the maximization of succinate uptake. While surprising at first, this result is intuitive considering that, to maximize growth, the cell needs to maximize uptake of its limiting nutrient. This simple example already points out an important aspect of FBA and its inverse problem, as addressed by invFBA: while the inverse algorithm rules out a large subset of objectives whose optimization could not possibly lead to the observed fluxes, different **c** vectors may still, when used in FBA, yield the same observed fluxes. Note that if two such equivalent objectives were used in the forward FBA problem, it is not guaranteed they will produce the same fluxes, due to the existence of alternative optimal solutions in FBA itself. Yet, any **c** inferred by invFBA will produce a flux distribution lying on the facet of the FBA polyhedron, which contains all optimal flux distributions. While the above analysis was focused on testing the capacity of invFBA to recover growth maximization as the underlying objective, one may wonder whether the algorithm could similarly recover alternative objectives. Towards this goal, we generated FBA-predicted fluxes using maximization of ATP synthase flux and minimization of glucose uptake for a fixed growth rate as alternative objectives. As shown in Additional file 6: Table S8 and Additional file 7: Table S9, the sparse invFBA algorithm consistently recovered the correct objective function.

### Recovering objectives and fluxes from noisy data

**Fig. 3:**
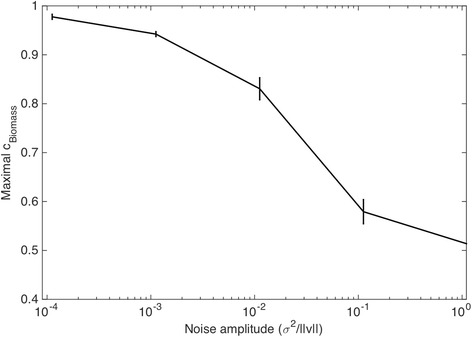
Robustness of invFBA to noise. The maximal value of the biomass coefficient c_biomass_, found by invFBA and subsequent objective variability analysis (OVA), is plotted as a function of the level of noise (*σ* ^*2*^) in FBA-simulated flux data for *E. coli*. These FBA-simulated fluxes are produced using maximization of biomass production as the objective function. Thus, a value of c_biomass_ close to unity in OVA indicates that invFBA recovers the original objective. As the level of noise increases, however, our ability to recover the original objective is highly reduced

Unlike fluxes predicted by FBA simulations, experimentally observed fluxes will likely contain some noise that may mask the compatibility with different optimality criteria. For example, while any FBA flux vector predicted through the maximization of the biomass flux will have precisely the maximal possible growth flux value, experimentally measured fluxes, even if close to a growth optimum, will likely fall within an area around it. In order to simulate this process and test invFBA under noisy flux measurements, we implemented our inverse algorithm under increasing levels of noise and tested our capacity to recover the correct objective. In particular, we wanted to add noise to the optimal solution of FBA while keeping noisy fluxes in the feasible solution space (i.e. so that all reactions are in steady state and mass-balanced). This can be achieved by running an additional FBA-like optimization that samples random points close (within a given radius *σ*^*2*^) to a previously computed FBA optimum (see Additional file 1: Supplementary Methods). As shown in Fig. 3, as the noise approaches zero, invFBA solutions converge to having as main component the growth maximization objective. As the magnitude of the noise increases, the maximum possible value for the biomass reaction component of the objective decays further and further away from unity, with a major downshift at the point where the noise level is between 1 % and 10 % relative to the flux norm. At that point, the information carried by the noisy fluxes is not informative of the original objective any more.

### Applying invFBA to time-dependent fluxes inferred through an integrated expression-FBA model

After testing the performance of invFBA on exact or noisy flux distributions simulated by FBA, we took a first step towards employing invFBA for the analysis of experimental data. In particular, we applied invFBA to genome-scale metabolic fluxes inferred from a combination of experimentally measured gene expression data and stoichiometric modeling. Collins et al. inferred putative time-series flux vectors from time-series gene expression data at different stages of growth by *Shewanella oneidensis* under aerobic, carbon-limited conditions [12]. The method used for that analysis (temporal expression-based analysis of metabolism [TEAM] [12]), extended a prior approach [13] by penalizing the cost of maintaining flux through a reaction with low gene expression. In TEAM, in contrast to prior methods, the penalty, different for each gene, was estimated based on a large compendium of gene expression data. Like many other FBA-gene expression integration methods, TEAM does not use a biologically motivated pre-assumed objective function, but rather maximizes consistency with measured gene expression data. Thus, fluxes inferred through TEAM correspond to the outcome of a heuristic approach for the interpretation of expression data in terms of metabolic fluxes, but do not assume any prior knowledge on the metabolic objective of the cell. We should emphasize that, as described recently [14], integration of gene expression data to help predict fluxes is still problematic, partly due to the non-trivial relationship between mRNA and protein levels [15, 16]. However, in the context of the current work, the TEAM-inferred dataset gives the unique opportunity of obtaining putative objectives from genome-scale fluxes that reflect the metabolic effort of the bacterium as it undergoes changes throughout batch growth.

**Fig. 4:**
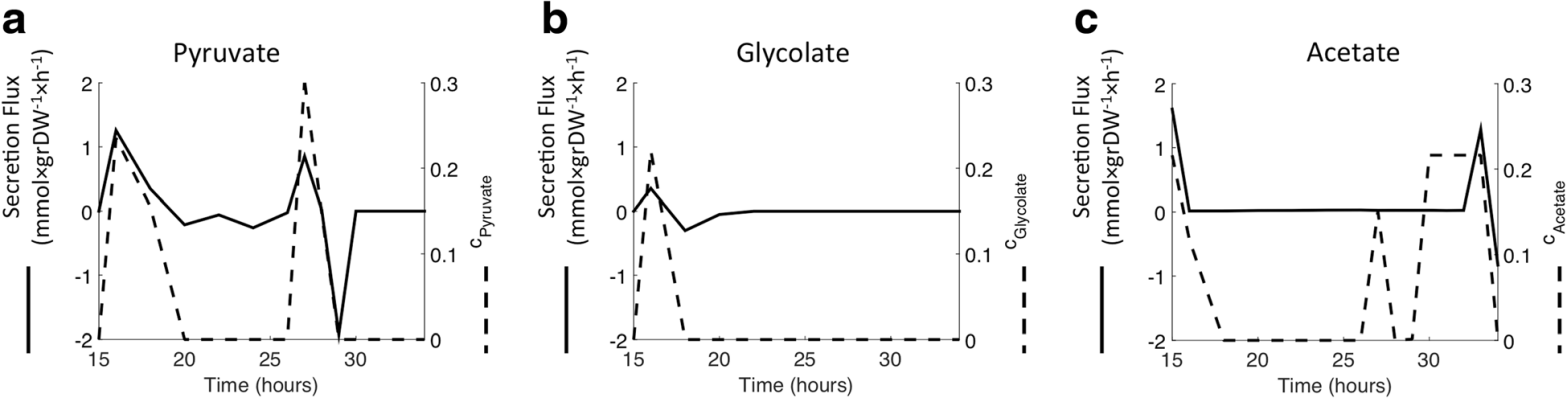
Comparison of invFBA predictions and metabolite secretion. Metabolite secretion flux (*full line*), inferred from experimental data through the TEAM approach, and maximum coefficient of the secretion flux in the objective function (*dashed line*), as predicted by invFBA (through OVA), are plotted as a function of time. Metabolite and gene expression data come from time-dependent measurements performed during batch aerobic growth of the bacterium *S. oneidensis* on lactate. The secreted metabolites are pyruvate in (**a**), glycolate in (**b**), and acetate in (**c**). Positive fluxes reflect secretion of the metabolite in question, while negative fluxes reflect uptake

As in the previous case of model-generated fluxes, the inverse problem admits a large space of possible solutions, i.e. maximally sparse objective functions that could give rise to the observed fluxes. Rather than providing specific arbitrary choices of objectives within the possible range, we report the outcome of OVA, as described above. Among all possible components of the identified objectives, we highlight the ones that can be compared directly with non-trivial experimental flux measurements, e.g. pyruvate secretion/uptake. The scope of OVA, or, more precisely, the reactions it can include in the objective function, was accordingly confined to exchange reactions. As seen in Fig. 4a, the largest pyruvate secretion component of the objective function (as computed by OVA) at different time points recapitulates the experimentally detected accumulation of pyruvate in the external medium, previously hypothesized to be the outcome of overflow metabolism [17, 18]. The same trend holds for glycolate (Fig. 4b) and acetate (Fig. 4c), although invFBA predicts optimization of acetate secretion at several time points leading up to the renewed secretion of acetate at 33 h. Applied to genome-scale fluxes obtained at each sample along the growth curve, the integer-programming variant of invFBA (see Additional file 1: Supplementary Methods) identified biomass production as the objective function at all time points. Optimization of biomass production agrees with these flux distributions originating from a growing *S. oneidensis* culture [8, 12]. These results lend confidence to the capacity of invFBA and OVA to correctly capture essential features of flux datasets. At the same time, they highlight the importance of being cautious in the interpretation of objective functions, as a large component of the objective (e.g. pyruvate secretion) cannot be necessarily ascribed to a specifically evolved metabolic trait and may rather be the outcome of undesirable overflow metabolism.

### Inference of objective functions in *E. coli* strains that underwent long-term evolutionary experiments

The most interesting application of invFBA is the inference of objective functions for microbial species and environments for which direct flux measurements are available and important questions on adaptation and optimality are at stake. An excellent example of this scenario is the availability of recently measured metabolic flux ratios [6] for some of the *E. coli* strains that underwent long-term experimental evolution in the Lenski Lab. These strains were evolved for 50,000 generations in glucose minimal medium, leading to important observations and discoveries on how adaptation works [19–22]. The reported flux ratios can be converted to flux vectors compatible with the stoichiometric constraints (see Additional file 1: Supplementary Methods).

**Fig. 5:**
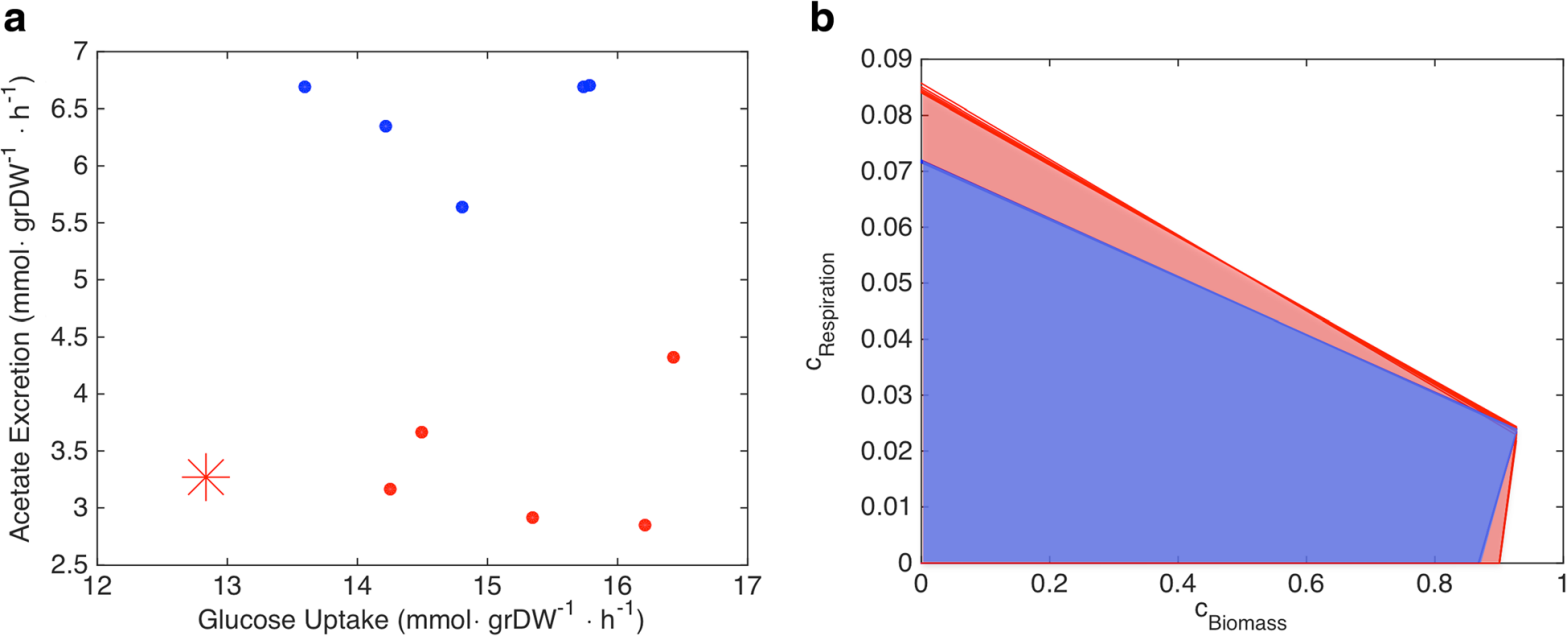
Application of invFBA to long-term evolved *E. coli* strains. **a** Experimental measurements (by Harcombe et al. [6]) of acetate excretion and glucose uptake for the ancestral (*red star*) and evolved (*blue and red dots*) *E. coli* strains from Lenski’s long-term evolutionary experiment. The *red* and *blue* colors are used here to highlight two distinct metabolic regimes that different strains seem to cluster around. **b** A *projection* (onto a two-dimensional subspace) of the set of objective functions compatible with experimentally measured fluxes. The *graph* is obtained through a two-dimensional version of OVA: for each possible value of the growth flux coefficient of the objective function (c_Biomass_), one can find the minimal and maximal value of the objective function coefficient for the respiratory flux (c_Respiration_), obtaining areas that correspond to objective functions compatible with the measured fluxes. Such regions can be computed for the ancestral and all evolved *E. coli* strains. The strains corresponding to the different metabolic regimes (*blue and red dots* in **a**) map onto different regions in the space of objectives, labeled with similar colors

The previous FBA analysis of metabolic activity in these strains had suggested that objective functions other than standard biomass flux maximization may best describe their evolutionary trajectory [6]. Such analysis, however, only assessed the capacity of a small set of specific objective functions to lead to correct fluxes. Using invFBA, it is possible to reanalyze these flux data in an unbiased way and characterize the space of objective functions compatible with the observations. A particularly striking feature of the flux data was the fact that six of the strains (five evolved and the ancestral) show comparatively low levels of acetate secretion (or, equivalently, high levels of glucose oxidation), as illustrated in Fig. 5a.

Upon applying invFBA to the complete set of measured flux data (Additional file 8: Table S4 and Additional file 9: Table S5), we found, as in the aforementioned case studies, an infinite set of possible solutions, i.e. a convex polyhedral set of objective functions. Each of these objective functions – if used in FBA – could give the observed fluxes as an optimal solution (one of many alternative optima). Interestingly, the objective function consisting of only maximization of growth flux is not part of any of these sets (neither for the ancestral, nor for the evolved). Using OVA, one can find the maximal possible contribution of the growth flux in the objective function (Additional file 10: Table S6). While OVA provides ranges for the contributions to the objective by individual fluxes, it does not give any information on correlations and tradeoffs between the different flux components in the objective function. Visualization of the whole space of possible objective functions identified by invFBA is possible only upon reducing somehow the dimensionality. As illustrated in Fig. 5b, this can be achieved by projecting the space of possible optimal objectives onto a two-dimensional plane whose components are two specified biologically interesting fluxes. Upon visualizing this space in the plane of growth versus respiration flux, again two sets of strains readily emerge, corresponding to the low- and high-respiration strains shown in Fig. 5b. For the same objective coefficient in the growth reaction, the low-acetate secreting strains have a lower maximal objective coefficient for respiration (in a way that is robust to experimental error as shown in Additional file 11: Figure S1). This means that, despite the freedom of choice of objectives compatible with the experimental data, the signatures of how different fluxes may have adapted are still readable from the specific boundaries of the space of feasible objectives. Our analysis captures the dichotomy observed at the level of acetate and glucose transport fluxes and suggests that the low acetate-excretion strains may be interpreted as having a higher maximization of respiration.

## Discussion

To identify the most fitting objective function for a given set of observed fluxes, we formulated invFBA as an optimization problem whose linearity guarantees global optimality and polynomial computation. In general, there exists no unique solution to the invFBA optimization. For example, to optimize growth, the cell must simultaneously optimize other reactions, like uptake of the limiting nutrient. Even with additional heuristics (such as sparsity or regularization), it may be impossible to identify a single appropriate objective; instead a whole set of objectives can be equally compatible with the data. It is important to note that this is a fundamental theoretical limit associated with linear optimization-based metabolic network models in general, rather than a limit in algorithmic capabilities. One possible interesting application of this uncertainty might be in the field of metabolic engineering, where it is often difficult to specifically evolve a desired trait. One could envisage that, using a variant of our invFBA framework, it would be possible to search for pairs of objectives such that maximization of one would likely also yield optimization of the other.

While the large space of possible objectives compatible with almost any flux distribution is intrinsic to the complex nature of metabolic networks, it presents a challenge to the biological insight potentially gleaned from invFBA. Inducing sparsity through integer programming readily collapses the space of optimality into a few objective functions with clear biological meaning, as shown in our TEAM analysis (Fig. 4). Alternatively, generating flux distributions in FBA with objectives sampled from the large space of optimality outlined by invFBA, then comparing these computed fluxes with observed fluxes might enable us to associate with each objective a certain probability and subsequently select the most likely objective (not unlike the work in [10]). Furtermore, flux coupling analysis [23], an established method in the literature, could serve as a means to group together closely related metabolic reactions, thereby reducing the dimensionality of the objective space captured by invFBA. Within the boundaries of the fundamental uncertainty quantifiable with OVA, invFBA succeeds in recovering the true underlying objective function from simulated fluxes, among less biologically meaningful, albeit equally optimal objectives. With simulated data, the true solution was known *a priori*, since the “observed fluxes” were calculated in FBA by optimizing biomass production. The addition of different degrees of noise allowed us to estimate the level of experimental error in the measurements beyond which information about the underlying objective function is unrecoverable. The real relevance of invFBA is, however, in the capacity to receive as input actual experimental data, inherently noisy, and pertaining to biological systems for which the true solution (i.e. the objective being optimized) is unknown. Future inverse problems would benefit from analysis under different conditions, in order to find objectives that are common to the different conditions.

Our inverse algorithm can be particularly useful for analyzing evolutionary experiments, where the objective functions can be thought of as a high-level representation of targets of selection, which can be inferred based on flux measurements. Here, we exemplified this concept by applying our approach to measured fluxes from the *E. coli* “Lenski lines” [19–22]. While all strains evolved faster rates of glucose uptake, which largely accounts for their greater growth rates, six strains further optimized respiratory efficiency, consistent with measurements of lower acetate secretion (alternatively, higher efficiency of glucose oxidation). The well-known trade-off between metabolic rate and yield allows fermentative yeast to outcompete other single-cellular organisms by rapidly exhausting a carbon source. One might speculate that low-respiration Lenski strains, much like yeast, favor a high-rate strategy at the expense of yield, whereas high-respiration strains favor a high-yield strategy at the expense of rate. However, Fig. 5a shows rates of glucose uptake to be comparable between both sets of evolved *E. coli* populations, with means of 14.8 and 15.3 mmol/grDW*h for high- and low-respiration strains, respectively. Low yields of glucose utilization do not, therefore, confer high rates of glucose uptake to evolved populations of *E. coli*. One way to interpret our results is to consider that evolving faster means of glucose uptake most likely overwhelms enzymes responsible for glucose breakdown and energy conservation. As a result, in strain REL1, for instance, the rate of acetate secretion amounts to almost half the rate of glucose consumption. Evidently, while all *E. coli* populations evolved faster glucose uptake, only half were also able to adapt their catabolic enzymes to efficiently utilize additional glucose. As seen in Fig. 5a, only high-respiration populations could bring down their acetate production to glucose uptake ratio to levels seen in the ancestral strain. Thus, invFBA gives interpretable results for experimental fluxes, without the benefit of prior knowledge concerning the true underlying objective of the cell.

Interpreting a set of fluxes belonging to a metabolic model with hundreds of reactions is a nontrivial mathematical task, and our present formulation of invFBA lays the groundwork for future efforts in trying to infer cell-level goals from flux measurements. While efficient and overall capable of providing biological insight, invFBA still carries some of the limitations inherent in the definition of a linear metabolic objective function. In particular, as argued before [24], the weighted sums of reaction fluxes, inferred as putative objective functions, are not necessarily easily interpretable in terms of biological processes. This is in contrast to the classical LP example from economics, in which weights often represent the costs of different products of processes and the optimization seeks to minimize a linear combination that amounts to total expenses. By virtue of a common unit of currency, the weighted sum (or cost function) adds up to a number, in dollars, with intuitive meaning. The weighted sum becomes difficult to interpret quantitatively in the context of metabolic models, since different reactions do not necessarily use the same “currency.” Interestingly, however, the weighted sum can be mapped onto the space of Pareto optimality, whose interpretation is well-understood [3, 5, 25], tracing the boundary between two competing objectives where one can increase only at the expense of the other. Notably, as pointed out in [24], weighted sums capture the Pareto frontier only when said frontier is convex, which may not be, in general, the case within metabolic models.

An additional factor to take into consideration when interpreting the weights computed by invFBA for different fluxes contributing to the objective function is the possibility of biases and inaccuracies merely due to the wide range of magnitudes of different fluxes in a model. The growth reaction, for example, typically assumes values on the order of 1 h^–1^, while the glucose uptake is often about 20 mmol/(gDW*h). An objective function built as a linear combination of these two fluxes with equal weights would tend to skew the results towards maximizing glucose uptake, just due to the higher numerical reward of the ensuing solution. One would have to heavily weight the growth flux to see any flux through this reaction, resulting conversely in a skewed distribution of weights. The specific values of the weights should therefore not be necessarily considered as reflecting the importance of the respective fluxes in the objective. The typical numerical magnitude of the involved fluxes should be taken into account when attempting biological interpretations of the objective function coefficients. For example, in our study of evolved *E. coli* strains from the Lenski Lab, two fully coupled reactions belonging to the same linear metabolic pathway, identified in the small *E. coli* model [26] by flux coupling analysis [23], only give degenerate solutions in the space of possible objective functions provided they share the same numerical flux value (e.g. transketolase and transaldolase in the pentose phosphate pathway, Additional file 12: Figure S2). In interpreting invFBA results one should also keep in mind that wide differences in the characteristic values of different fluxes may favor specific solutions. These problems are similar to issues commonly raised and discussed in other flux balance modeling methods, such as the weights of different fluxes in MOMA [5], parsimonious FBA [27], and crowding-dependent FBA [28]. Reformulating the problem with normalized variables could alleviate this issue, but at the expense of increased problem complexity and non-linearity. For example, normalizing objective coefficients by the number of carbon atoms at play in any given reaction might allow a fair comparison across all weights calculated through invFBA.

A last important caveat about the current formulation of invFBA is that it requires a complete flux distribution as input. Given that ^13^C-labeled nutrient experiments can usually only quantify fluxes for a small number of reactions in central carbon metabolism, invFBA from experimental flux measurements would only be possible for reduced stoichiometric models, like the one used in our analysis of the “Lenski lines” [6]. For these types of experimental data, future versions of invFBA could infer objectives directly from the measured flux ratios, thereby minimizing the chance of propagation of errors across multiple algorithms.

## Conclusions

While in stoichiometric modeling the space of feasible cellular states has been explored extensively using a number of formal approaches, including optimality, sampling, and convex geometry theory, much less theoretical work has been invested in exploring the space of possible objective functions. Most attempts at exploring this space have been hampered by computational complexity or limited to empirical comparison of a few specific choices of alternative objective functions. Our new inverse objective-finding algorithm establishes a theoretical framework that will enable a more formal, efficient, and systematic analysis of the space of possible objective functions. The framework, currently limited to linear objectives, could be extended to non-linear objectives and to alternative strategies for regularization, possibly introducing additional biological constraints.

Finally, the approach developed for invFBA constitutes a specific instance of a broader, powerful, and still highly unexplored avenue for posing and solving inverse optimization problem. The relevance of our algorithm may extend beyond the realm of metabolic network modeling, for example to game-theoretic models of traffic equilibria in transportation and price-setting games in economics.

## Methods

### Flux balance analysis (FBA)

To mathematically formulate FBA, let **S** denote the stoichiometric matrix of dimensions *m* x *n* where *m* is the number of metabolites and *n* the number of metabolic fluxes, **x** the vector of metabolic fluxes (internal and external), **c** the vector of coefficients expressing the cellular objective (e.g. biomass), Z_opt_ the optimal objective value, and **x**_***lb***_*,***x**_***ub***_ lower and upper bounds, respectively, on the metabolic fluxes, implied by empirical evidence of irreversibility or by nutrient availability in growth medium [1]. The FBA problem is formulated as:

1 $$ \begin{array}{ll}{Z}_{opt}=\hfill & ma{x}_{\mathbf{x}}\kern0.5em \boldsymbol{c}\boldsymbol{\hbox{'}}\mathbf{x}\hfill \\ {}\hfill & \mathrm{s}.\mathrm{t}.\kern0.75em \mathbf{S}\mathbf{x}=0,\hfill \\ {}\hfill & {\mathbf{x}}_{\boldsymbol{lb}}\le \mathbf{x}\le {\mathbf{x}}_{\boldsymbol{ub}},\hfill \end{array} $$

where **0** is the vector of all zeroes and primes indicates transpose.

### Inverse flux balance analysis (invFBA)

Let us assume we have a set of *N* measured metabolic flux vectors **x**_*i*_, where *i = 1,…,N*. Let us also assume that, due to measurement noise, these flux vectors are not necessarily optimal for a specific objective, even if they are feasible solutions of the FBA problem (Eq. 1). With x* denoting an optimal solution of Eq. 1, let *ϵ*_*i*_ ≥ 0 denote the suboptimality gap of **x**_*i*_, i.e. the distance between the measured objective function value and the predicted one. This implies:

$$ {\mathbf{c}}^{\prime }{\mathbf{x}}^{\ast }-{\boldsymbol{c}}^{\prime }{\mathbf{x}}_i={\epsilon}_i $$

The invFBA problem consists of finding a set of objective functions that minimize this suboptimality gap. Through duality theory (see Additional file 1: Supplementary Methods for details), the problem can be formulated as the following linear programming problem:

2 $$ \begin{array}{ll}{\mathrm{Z}}_{opt}^I=\hfill &\ { \min}_{{\boldsymbol{p}}^{\boldsymbol{i}},\ {\boldsymbol{q}}_1^{\boldsymbol{i}},\ {\boldsymbol{q}}_2^{\boldsymbol{i}},{\upepsilon}_{\boldsymbol{i}},\boldsymbol{c}}\kern0.75em {\displaystyle {\sum}_{i=1}^N{\epsilon}_i}\hfill \\ {}\hfill & \mathrm{s}.\mathrm{t}.{\displaystyle {\sum}_{j=1}^n{c}_j=1,}\hfill \\ {}\hfill & {\mathbf{p}}^{\mathbf{i}\hbox{'}}\mathbf{S} - {\boldsymbol{q}}_1^{{\boldsymbol{i}}^{\boldsymbol{\hbox{'}}}}+{\boldsymbol{q}}_2^{{\boldsymbol{i}}^{\boldsymbol{\hbox{'}}}}={\boldsymbol{c}}^{\boldsymbol{\hbox{'}}},\ \forall i,\hfill \\ {}\hfill & {\boldsymbol{q}}_2^{\boldsymbol{i}\boldsymbol{\hbox{'}}}{\mathbf{x}}_{\boldsymbol{ub}}-{\boldsymbol{q}}_1^{{\boldsymbol{i}}^{\boldsymbol{\hbox{'}}}}{\mathbf{x}}_{\boldsymbol{lb}}-{\epsilon}_i = {\boldsymbol{c}}^{\hbox{'}}{\mathbf{x}}_i,\ \forall i,\hfill \\ {}\hfill & {\boldsymbol{q}}_1^{\boldsymbol{i}},\kern0.75em {\boldsymbol{q}}_2^{\boldsymbol{i}}\ge 0,\forall i,\hfill \\ {}\hfill &\ {\epsilon}_i\ge 0,\forall i,\hfill \end{array} $$

where the second, third, and fourth constraint in the display above define the feasible space of objective functions compatible with the measured fluxes (a cone **C**), defined in terms of the dual variables **p**, **q**_1_, **q**_2_ (associated with the FBA problem in Eq. 1), the first constraints introduces a normalization which guarantees a non-zero solution, and *Z*^*I*^_*opt*_ denotes the minimal total suboptimality gap of the measured metabolic flux distributions **x**_*i*_.

We propose a subsequent step in invFBA to minimize the L1-norm of optimal **c** = (c_1_,…,c_n_) vectors obtained from solving the problem in Eq. 2:

3 $$ \begin{array}{l}{ \min}_{{\boldsymbol{p}}^{\boldsymbol{i}},\ {\boldsymbol{q}}_1^{\boldsymbol{i}\boldsymbol{\hbox{'}}},\ {\boldsymbol{q}}_2^{\boldsymbol{i}\boldsymbol{\hbox{'}}},{\upepsilon}_{\boldsymbol{i}},\boldsymbol{c}}{\displaystyle {\sum}_{j=1}^n\left|{c}_j\right|}\hfill \\ {}\mathrm{s}.\mathrm{t}.{\displaystyle {\sum}_{i=1}^N{\epsilon}_i={Z}_{opt}^I},\hfill \\ {}{\displaystyle {\sum}_{j=1}^n{c}_j=1,}\hfill \\ {}{\mathbf{p}}^{\mathbf{i}\hbox{'}}\mathbf{S} - {\boldsymbol{q}}_1^{{\boldsymbol{i}}^{\boldsymbol{\hbox{'}}}}+{\boldsymbol{q}}_2^{{\boldsymbol{i}}^{\boldsymbol{\hbox{'}}}}={\boldsymbol{c}}^{\boldsymbol{\hbox{'}}},\ \forall i,\hfill \\ {}{\boldsymbol{q}}_2^{\boldsymbol{i}\boldsymbol{\hbox{'}}}{\mathbf{x}}_{\boldsymbol{ub}}-{\boldsymbol{q}}_1^{{\boldsymbol{i}}^{\boldsymbol{\hbox{'}}}}{\mathbf{x}}_{\boldsymbol{lb}}-{\epsilon}_i = {\boldsymbol{c}}^{\hbox{'}}{\mathbf{x}}_i,\ \forall i,\hfill \\ {}{\boldsymbol{q}}_1^{\boldsymbol{i}},\kern0.75em {\boldsymbol{q}}_2^{\boldsymbol{i}}\ge 0,\forall i,\hfill \\ {}{\epsilon}_i\ge 0,\forall i.\hfill \end{array} $$

Part of the optimal solution of the problem in Eq. 3 is a sparse **c** vector that renders the given set of measured metabolic flux distributions **x**_1_,…,**x**_N_ near-optimal in the FBA optimization (Eq. 1). One can then interpret non-zero elements of **c** as corresponding to important metabolic fluxes that are critical in the FBA optimization context and provide a minimal description of the cellular objective function. In the sequel, when we refer to an invFBA algorithm, we mean the two-step procedure of solving the problems in Eqs. 2 and 3. Alternative regularization schemes are illustrated in the Additional file 1: Supplementary Methods and Additional file 13: Figure S3, and a formal proof of a theorem establishing that solutions **c** of invFBA guarantee each measured **x**_*i*_ to be near-optimal for the FBA (Eq. 1) can be found in [29]. Problem [8] is a linear programming problem minimizing the L1 norm of the vector **c**. It can be viewed as a convex relaxation of a problem with identical constraints, which minimizes the L0 norm of **c** (i.e. the number of non-zero elements in **c**). The latter problem can be formulated as an integer programming problem. For more details, please refer to the problem [S8.1] in Additional file 1: Supplementary Methods.

An important observation is that both problems in Eqs. 2 and 3 that comprise our invFBA algorithm are linear programming problems. This is important because it guarantees a global optimal solution (as opposed to earlier approaches as in [8] resulting in non-convex problems). Moreover, very efficient polynomial-time algorithms exist for solving such problems. It is interesting that the complexity of the invFBA algorithm matches that of FBA - both are linear programing problems, which, in general, is not true for inverse optimization problems [9, 10]. We note that the duality approach to inverse optimization has been used in a more general setting in [30]; however, to the best of our knowledge, ours is the first attempt to rigorously characterize the set of FBA objective functions consistent with a potentially noisy set of measurements.

### Availability of data and scripts

All data and scripts used to generate the figures presented in this work are available under an MIT open access license in a zipped directory at Figshare (https://dx.doi.org/10.6084/m9.figshare.3181504.v1). The directory contains subdirectories for specific figures and general algorithms (including the InvFBA function). The scripts are in Matlab (.m) and use datafiles in Matlab binary format (.mat). Paths pointing to the optimization software (e.g. Gurobi, which we used under a free academic license) need to be updated by the user.

### Ethical approval

No ethical approval was required for this study.

## Additional files

Additional file 1: A Supplementary Methods file describes details of the InvFBA algorithm. (PDF 9300 kb) Additional file 2: Table S7. shows invFBA results from L1 and L0 norm regularization of simulated *E. coli* data under different growth conditions (with growth flux maximization as the objective). (XLSX 8 kb) Additional file 3: Table S1. shows non-zero minimal/maximal optimization coefficients computed by objective variability analysis (OVA) for each reaction from simulated glucose-limited *E. coli* fluxes. (XLSX 9 kb) Additional file 4: Table S2. shows non-zero minimal/maximal optimization coefficients computed by objective variability analysis (OVA) for each reaction from simulated succinate-limited *E. coli* fluxes. (XLSX 9 kb) Additional file 5: Table S3. shows non-zero minimal/maximal optimization coefficients computed by objective variability analysis (OVA) for each reaction from simulated glycerol-limited *E. coli* fluxes. (XLSX 9 kb) Additional file 6: Table S8. shows invFBA results from L1 and L0 regularization of simulated *E. coli* data under different growth conditions (with maximization of ATP synthase as the objective). (XLSX 8 kb) Additional file 7: Table S9. shows invFBA results from L1 and L0 regularization of simulated *E. coli* data under glucose minimal medium (with minimization of glucose uptake as the objective). (XLSX 8 kb) Additional file 8: Table S4. shows all reactions in small *E. coli* model. (XLSX 9 kb) Additional file 9: Table S5. shows metabolic fluxes inferred from flux ratios for 11 evolved *E. coli* strains. (XLSX 13 kb) Additional file 10: Table S6. shows maximal optimization coefficients computed by objective variability analysis (OVA) for each reaction from fluxes measured in 11 evolved *E. coli* strains. (XLSX 12 kb) Additional file 11: Figure S1. shows the results of a robustness analysis of OVA-predicted optimization of respiration in evolved *E. coli* strains. (PDF 144 kb) Additional file 12: Figure S2. illustrates the application of invFBA to two fully coupled reactions in long-term evolved *E. coli* strains. (PDF 105 kb) Additional file 13: Figure S3. provides a schematic visualization of possible objective functions through invFBA, with L2-regularization. (PDF 182 kb)

## Abbreviations

FBA: Flux balance analysis
invFBA: Inverse flux balance analysis
LP: Linear programming
OVA: Objective variability analysis
TEAM: Temporal expression-based analysis of metabolism
