# Supplementary material for: Mapping the landscape of metabolic goals of a cell
Source: Genome Biol. 2016 May 23;17:109. doi: 10.1186/s13059-016-0968-2 (PMC4878026; doi:10.1186/s13059-016-0968-2)
Supplement: Additional file 1: — A Supplementary Methods file describes details of the InvFBA algorithm. (PDF 9300 kb) [file 13059_2016_968_MOESM1_ESM.pdf]

SUPPLEMENTARY METHODS

Flux Balance Analysis (FBA)

To mathematically formulate FBA, let **S** denote the stoichiometric matrix of dimensions *m* x *n* where *m* is the number of metabolites and *n* the number of metabolic fluxes, **x** the vector of metabolic fluxes (internal and external), **c** the vector of coefficients expressing the cellular objective (e.g., biomass), Z_{opt} the optimal objective value, and $\mathbf{x}_{lb}$, $\mathbf{x}_{ub}$ lower and upper bounds, respectively, on the metabolic fluxes implied by empirical evidence of irreversibility or by the composition of the growth medium. The FBA problem is formulated as:

$$\begin{aligned} Z_{opt} &= max_{\mathbf{x}}\ \boldsymbol{c}'\mathbf{x} \\ \text{s.t.}\ \ \mathbf{Sx} &= \mathbf{0}, \\ \mathbf{x}_{lb} \leq \mathbf{x} &\leq \mathbf{x}_{ub}, \end{aligned} \qquad [S1]$$

where **0** is the vector of all zeroes and primes indicates transpose.

Inverse Flux Balance Analysis (invFBA)

Let us assume we have a set of measured metabolic flux distributions $\mathbf{x}_i$, where $i = 1,...,N$. Let us also assume that, due to measurement noise, these flux distributions are not necessarily optimal, even if they are feasible solutions of the FBA problem (Eq. S1). With $\mathbf{x}^*$ denoting an optimal solution of Eq. 1, let $\epsilon_i \geq 0$ denote the suboptimality gap of $\mathbf{x}_i$, i.e., the distance between the measured objective function value and the predicted one. This implies:

$$\mathbf{c}'\mathbf{x}^* - \boldsymbol{c}'\mathbf{x}_i = \epsilon_i$$

It is well known that for every maximization linear programming problem like Eq. 1 we can write a corresponding Lagrangian dual problem (24):

$$\begin{aligned} &\min_{p,q_1,q_2}\ \boldsymbol{q}_2'\mathbf{x}_{ub} - \boldsymbol{q}_1'\mathbf{x}_{lb} \\ &\text{s.t.}\ \ \mathbf{p}'\mathbf{S} - \boldsymbol{q}_1' + \boldsymbol{q}_2' = \boldsymbol{c}', \\ &\quad\ \boldsymbol{q}_1,\ \boldsymbol{q}_2 \geq \mathbf{0}, \end{aligned} \qquad [S2]$$

where $\boldsymbol{p}$, $\boldsymbol{q}_1$, and $\boldsymbol{q}_2$ are the dual variables corresponding to the constraint $\mathbf{Sx} = \mathbf{0}$, $\mathbf{x} \geq \mathbf{x}_{lb}$ and $\mathbf{x} \leq \mathbf{x}_{lb}$ respectively. The optimal objective values of (the primal) problem (Eq. S1) and the dual problem (Eq. S2) are equal (strong duality (24)). Each element p_i, i=1,…, m of $\boldsymbol{p}$ can be interpreted as a shadow price for metabolite i. Assuming that none of the inequality constraints in Eq. S1 are binding, there exists a set of prices $\boldsymbol{p}$ so that the value c_k of one unit of flux x_k^* is equal to the sum of the prices of the metabolites that make up x_k in the proportions given by the kth column of the stoichiometry matrix **S**.

For any measured (and feasible) metabolic flux distribution $\mathbf{x}_i$, and using Eq. S2, the strong duality theorem (which ensures the equality of the optimal primal and dual objectives) [25] implies the following set of conditions:

$$\begin{aligned} &\mathbf{p}^{i\prime}\mathbf{S} - \boldsymbol{q}_1^{i\prime} + \boldsymbol{q}_2^{i\prime} = \boldsymbol{c}', \forall \boldsymbol{i}, \\ &\quad \boldsymbol{q}_2^{i\prime}\mathbf{x}_{ub} - \boldsymbol{q}_1^{i\prime}\mathbf{x}_{lb} - \epsilon_i = \boldsymbol{c}'\mathbf{x}_i, \forall i, \\ &\quad \boldsymbol{q}_1^i,\ \boldsymbol{q}_2^i \geq \mathbf{0}, \forall i. \end{aligned} \qquad [S3]$$

These equations guarantee that $\mathbf{x}_i$ is near-optimal, that is, its objective cost is ϵ_i away from the optimal objective cost value of Eq. 1. Put differently, the equations above describe a set of vectors **c** that lead to the near-optimality of $\mathbf{x}_i$. This set of **c**'s is a cone **C**, namely, a set that contains all non-negative multiples of its elements. This conclusion is quite intuitive since it suffices to determine the objective coefficient vector up to a non-negative multiplicative constant. Fig. 1 illustrates the conic structure of the set of **c**'s that validate the optimality of a given metabolic flux distribution.

So far, we have characterized a set of **c** vectors that are consistent with the measurements $\mathbf{x}_i$. It is important to note that there are many valid cellular objectives (an infinite number of **c** vectors in the cone **C**) that are consistent with the measured flux distribution. Essentially, FBA modeling cannot lead to unique inference of the cellular objective from measured fluxes. Collecting the constraints in Eq. S3 for all measurements $i = 1,\ldots,N$ and minimizing the total sub-optimality gap, we obtain the following optimization problem (see Appendix A for further details):

$$\begin{aligned} \min_{\boldsymbol{p^i},\boldsymbol{q_1^i},\boldsymbol{q_2^i},\epsilon_i,\boldsymbol{c}} \ & \sum_{i=1}^{N} \epsilon_i \\ \text{s.t.} \quad & \mathbf{p}^{i\prime}\mathbf{S} - \boldsymbol{q_1^{i\prime}} + \boldsymbol{q_2^{i\prime}} = \boldsymbol{c}', \forall i, \\ & \quad \boldsymbol{q_2^{i\prime}}\mathbf{x_{ub}} - \boldsymbol{q_1^{i\prime}}\mathbf{x_{lb}} - \epsilon_i = \boldsymbol{c}'\mathbf{x}_i, \forall i, \\ & \boldsymbol{q_1^i},\ \boldsymbol{q_2^i} \geq \mathbf{0}, \forall i, \\ & \epsilon_i \geq 0, \forall i. \end{aligned} \qquad [S4]$$

We can interpret the above linear program as seeking a vector **c** that makes all measurement flux vectors $\mathbf{x}_i$ as close as possible to optimal flux distributions in the FBA problem (Eq. S1).

The problem in Eq. 4 has the trivial solution $\mathbf{c} = \mathbf{0}$, which is reasonable given our observation that we can only determine the objective coefficient vector up to a non-negative multiplicative constant. It follows that we need to introduce some form of *regularization* to restrict $\boldsymbol{c}$ to non-trivial choices.

One possible regularization is to add to the formulation in Eq. S4 the L2-norm equality constraint $||\mathbf{c}||^2 = 1$. In this case, the objective coefficient vector **c** lies on the surface of the unit ball in R^n. To gain more geometric insight into the proposed L2-regularized invFBA, consider the case of a single measured flux vector, say **x** (i.e., N = 1). Solving the problem in Eq. 4 amounts to minimizing $\mathbf{c}'(\mathbf{x}^j - \mathbf{x})$ over all **c** and all extreme points $\mathbf{x}^j$ of the FBA polyhedron (feasible set of Eq. 1). We have

$$\min_j \boldsymbol{c}'(\mathbf{x}^j - \mathbf{x}) = \min_j ||\boldsymbol{c}|| \, ||\mathbf{x}^j - \mathbf{x}|| \cos\alpha, \qquad [S5]$$

where α is the angle between **c** and $\mathbf{x}^j - \mathbf{x}$ and $||\mathbf{x}^j - \mathbf{x}|| \cos\alpha$ is the projection of $\mathbf{x}^j - \mathbf{x}$ onto **c**. Thus, and since $||\mathbf{c}|| = 1$, minimizing Eq. S5 over **c** is equivalent to projecting **x** on all facets of the FBA polyhedron and selecting the **c** that is perpendicular to the closest facet. As an example, in Fig. S3 (right), we compare the distances d_j, j = 1,…,4, between **x** and the four facets, which yields d_1 as the minimum and sets the corresponding optimal objective coefficient vector to $\mathbf{c}_1$.

Solving the FBA problem in Eq. S1, in practice, often leads to multiple optimal solutions. To select a unique optimal solution from the optimal solution set, a second optimization is required. In particular, one

minimizes the L1-norm of the metabolic flux distribution subject to the constraints of Eq. S1 and an additional constraint that guarantees the same objective value is achieved. The formulation is:

$$\begin{aligned} &\min_{\mathbf{x}} |\mathbf{x}| \\ \text{s.t. } &\boldsymbol{c}'\mathbf{x} = Z_{opt}, \\ &\mathbf{Sx} = \mathbf{0}, \\ &\mathbf{x}_{lb} \leq \mathbf{x} \leq \mathbf{x}_{ub}. \end{aligned} \qquad [S6]$$

An L1-norm is appealing because of its sparsity-inducing properties, which can help the biological interpretation of the solution. An L1-norm constraint can also be seen as a relaxation of the combinatorial problem that minimizes the number of nonzero elements of **c** in Eq. S4. In the same spirit, we propose the regularization constraint $\sum_{j=1}^{n} c_j = 1$ and add it to Eq. S4, which leads to the formulation:

$$\begin{aligned} Z_{opt}^{I} = \ &\min_{\boldsymbol{p}^i, \boldsymbol{q}_1^i, \boldsymbol{q}_2^i, \epsilon_i, \boldsymbol{c}} \sum_{i=1}^{N} \epsilon_i \\ &\text{s.t. } \textstyle\sum_{j=1}^{n} c_j = 1 \\ &\mathbf{p}^{\mathbf{i}\prime}\mathbf{S} - \boldsymbol{q}_1^{i\prime} + \boldsymbol{q}_2^{i\prime} = \boldsymbol{c}', \forall i, \\ &\qquad \boldsymbol{q}_2^{i\prime}\mathbf{x}_{ub} - \boldsymbol{q}_1^{i\prime}\mathbf{x}_{lb} - \epsilon_i = \boldsymbol{c}'\mathbf{x}_i, \forall i, \\ &\boldsymbol{q}_1^i, \ \boldsymbol{q}_2^i \geq \mathbf{0}, \forall i, \\ &\epsilon_i \geq 0, \forall i, \end{aligned} \qquad [S7]$$

where Z^{I}_{opt} denotes the optimal value.

Again motivated by the second step of FBA in Eq. S6, we propose a subsequent step in invFBA to minimize the L1-norm of $\mathbf{c} = (c_1, \ldots, c_n)$ vectors that solve Eq. S6:

$$\begin{aligned} &\min_{\boldsymbol{p}^i, \boldsymbol{q}_1^{i\prime}, \boldsymbol{q}_2^{i\prime}, \epsilon_i, \boldsymbol{c}} \sum_{j=1}^{n} |c_j| \\ &\text{s.t. } \textstyle\sum_{i=1}^{N} \epsilon_i = Z_{opt}^{I}, \\ &\textstyle\sum_{j=1}^{n} c_j = 1 \\ &\mathbf{p}^{\mathbf{i}\prime}\mathbf{S} - \boldsymbol{q}_1^{i\prime} + \boldsymbol{q}_2^{i\prime} = \boldsymbol{c}', \forall i, \\ &\qquad \boldsymbol{q}_2^{i\prime}\mathbf{x}_{ub} - \boldsymbol{q}_1^{i\prime}\mathbf{x}_{lb} - \epsilon_i = \boldsymbol{c}'\mathbf{x}_i, \forall i, \\ &\boldsymbol{q}_1^i, \ \boldsymbol{q}_2^i \geq \mathbf{0}, \forall i, \\ &\epsilon_i \geq 0, \forall i. \end{aligned} \qquad [S8]$$

Part of the optimal solution of Eq. S8 is a sparse **c** vector that renders the given set of measured metabolic flux distributions $\mathbf{x}_1, \ldots, \mathbf{x}_N$ near-optimal in the FBA optimization (Eq. S1). One can then interpret non-zero elements of **c** as corresponding to important metabolic fluxes that are critical in the FBA optimization context and provide a minimal description of the cellular objective function. In the sequel, when we refer to the *invFBA algorithm,* we mean the two-step procedure of solving problems Eq. S7 and Eq. S8. Due to the L1 regularization and the use of multiple flux vectors $\mathbf{x}_i$ as inputs to *invFBA*, the resulting **c** may not be perpendicular to one of the hyperplanes defining the FBA polytope; it can in fact be interior to the cone **C** containing all valid **c**'s.

Problem [S8] is a linear programming problem and it can be viewed as the convex relaxation of a problem that minimizes the L0-norm of **c** (i.e., the number of nonzero elements of **c**) instead of minimizing the L1-

norm of **c**. To pursue more sparse objective functions, an integer programming problem is introduced to minimize the L0-norm of **c**. This problem is formulated as:

$$\begin{aligned}
&\min_{\boldsymbol{p^i},\boldsymbol{q_1^{i\prime}},\boldsymbol{q_2^{i\prime}},\boldsymbol{\epsilon_i},\boldsymbol{c},\boldsymbol{z}} \sum_{j=1}^{n} z_j \\
&\text{s.t. } \sum_{i=1}^{N} \epsilon_i = \mathrm{Z}_{opt}^{I}, \\
&\sum_{j=1}^{n} c_j = 1, \\
&z_j \geq \frac{|c_j|}{L}, z_j \in \{0,1\}, \forall j, \\
&\mathbf{p^{i\prime}S} - \boldsymbol{q_1^{i\prime}} + \boldsymbol{q_2^{i\prime}} = \boldsymbol{c}', \forall i, \\
&\boldsymbol{q_2^{i\prime}}\mathbf{x_{ub}} - \boldsymbol{q_1^{i\prime}}\mathbf{x_{lb}} - \epsilon_i = \boldsymbol{c}'\mathbf{x}_i, \forall i, \\
&\boldsymbol{q_1^{i}},\ \boldsymbol{q_2^{i}} \geq \mathbf{0}, \forall i, \\
&\epsilon_i \geq 0, \forall i.
\end{aligned} \qquad \text{[S8.1]}$$

In [S8.1], the binary variable z_j is the indicator of whether c_j is nonzero (then $z_j = 1$) or not (then, $z_j = 0$). The constant L is a large number that together with the integrality constraint $z_j \in \{0,1\}$ forces z_j to be 1 when c_j is nonzero. Formulation [S8.1] is an integer programming problem and formulation [S8] is a convex relaxation of [S8.1]. Of course, it is much more computationally expensive to solve [S8.1] compared to solving [S8]. We can, however, use the solution of [S8] as a feasible solution for [S8.1], which can substantially speed up the solution time of [S8.1] (using the value of the [S8] solution in a branch-and-bound algorithm for [S8.1]). The results from [S8] and [S8.1] are shown in Table S7, S8 and S9.

LASSO version of invFBA

Here we provide an alternative LASSO version of invFBA that can be used instead of Eq. S7 and Eq. S8. The key idea is to add a sparsity-inducing L1 penalty for $\boldsymbol{c}$ in the objective. The formulation is:

$$\begin{aligned}
\mathrm{Z}_{opt}^{I} = &\min_{\boldsymbol{p^i},\boldsymbol{q_1^{i}},\boldsymbol{q_2^{i}},\boldsymbol{\epsilon_i},\boldsymbol{c}} \sum_{i=1}^{N} \epsilon_i + \lambda|\boldsymbol{c}| \\
&\text{s.t. } \sum_{j=1}^{n} c_j = 1 \\
&\mathbf{p^{i\prime}S} - \boldsymbol{q_1^{i\prime}} + \boldsymbol{q_2^{i\prime}} = \boldsymbol{c}', \forall i, \\
&\boldsymbol{q_2^{i\prime}}\mathbf{x_{ub}} - \boldsymbol{q_1^{i\prime}}\mathbf{x_{lb}} - \epsilon_i = \boldsymbol{c}'\mathbf{x}_i, \forall i, \\
&\boldsymbol{q_1^{i}},\ \boldsymbol{q_2^{i}} \geq \mathbf{0}, \forall i, \\
&\epsilon_i \geq 0, \forall i,
\end{aligned} \qquad \text{[S9]}$$

where λ is a tunable parameter that controls the sparsity of **c**.

Objective Variability Analysis

To analyze the variability of each element in the objective function within the optimal solution space, we developed a method called Objective Variability Analysis (OVA). OVA is a heuristic optimization method used to compute the upper and lower bound of the elements in the objective function. The first step of OVA is to solve the linear optimization problem given in Eq .S8.

By solving this problem, we can obtain an optimal solution for **c** and the optimal objective value $\sum_{i=1}^{N} \epsilon_i = \mathrm{Z}_{opt}^{I}$. Let $\mathcal{R}$ be the set of reaction indexes which are important in the FBA problem. To find the possible range for each c_r, r ϵ $\mathcal{R}$, we solve

$$
\begin{aligned}
\min_{\boldsymbol{p}^{i}, \boldsymbol{q}_1^{i}, \boldsymbol{q}_2^{i}, \epsilon_i, \boldsymbol{c}} \; & c_r + |\boldsymbol{c}| \\
\text{s.t.} \;\; & \textstyle\sum_{i=1}^{N} \epsilon_i = \mathrm{Z}_{opt}^{I} \\
& \textstyle\sum_{j=1}^{n} c_j = 1 \\
& \mathbf{p}^{\mathbf{i}'}\mathbf{S} - \boldsymbol{q}_1^{i'} + \boldsymbol{q}_2^{i'} = \boldsymbol{c}', \forall i, \\
& \boldsymbol{q}_2^{i'}\mathbf{x}_{\boldsymbol{ub}} - \boldsymbol{q}_2^{i'}\mathbf{x}_{\boldsymbol{lb}} - \epsilon_i = \boldsymbol{c}'\mathbf{x}_i, \forall i, \\
& \boldsymbol{q}_1^{i},\;\; \boldsymbol{q}_2^{i} \geq \mathbf{0}, \forall i, \\
& \epsilon_i \geq 0, \forall \boldsymbol{i},
\end{aligned}
\qquad \text{[S10]}
$$

and an identical problem with the only difference being that we maximize c_r **-** $|\boldsymbol{c}|$ (instead of minimizing) so as to find the largest possible value of c_r and maintain a small L1 norm of **c**. Solving these problems yields upper and lower bounds on each elements in the objective function.

To apply OVA in practice, some extra constraints on **c** should be added to Eq. S10. Consider reactions represented in the flux vectors $\mathbf{x}_i$ which have a flux equal to zero for all i. For these reactions, the corresponding elements c_j in **c** can take arbitrary feasible values because of the term $\boldsymbol{c}'\mathbf{x}_i$ in Eq. S10. For this reason, it is meaningless to run OVA on these c_j. To that end, we set $c_j=0$ for all those indices. In the case study involving simulated *E. coli* data, since the flux distributions are very sparse, we applied this technique and focused on the non-trivial reactions only.

Generation of noisy flux sets

In order to generate simulated feasible flux vectors around a defined point, containing a given amount of noise, we devised the following optimization problem:

$$
\begin{aligned}
& \max_{\mathbf{x}_i} \boldsymbol{r}'\mathbf{x}_i \\
& \text{s.t. } \boldsymbol{S}\mathbf{x}_i = \mathbf{0}, \\
& \quad \mathbf{x}_{\boldsymbol{lb}} \leq \mathbf{x}_i \leq \mathbf{x}_{\boldsymbol{ub}}, \\
& \quad ||\mathbf{x}_i - \mathbf{x}^*|| \leq \sigma^2,
\end{aligned}
$$

where $\boldsymbol{r}$ is a random objective function, $\mathbf{x}_i$ is the noisy flux distribution, $\mathbf{x}^*$ is the pre-computed optimal flux distribution, and σ^2 denotes the largest Euclidean distance between optimal flux and noisy flux. Changing the value of σ^2 yields different magnitudes of noise.

Inference of fluxes from experimentally measured branching ratios

To apply the invFBA algorithm and infer the objective function in *E. coli* strains that underwent long-term evolutionary experiments (LTEE), we needed to convert the ^{13}C-labeling raw measurements of flux ratios and uptake/secretion rates into central carbon metabolism flux values.

The dataset we used is obtained from Tables S1 (Growth parameters for ancestral and evolved LTEE isolates) and S2 (Experimentally determined flux ratios for ancestral and evolved LTEE isolates) from Harcombe WR, et al., PLoS Comput Biol 9(6): e1003091. This dataset includes measurements for one ancestral strain (Anc) and ten evolved strains (named, as in the original paper, A+1, A+2, A+3, A+4, A+5, A–1, A–2, A-4, A–5, A-6). For each strain, six pathway branch ratios (Ser from glycolysis, PYR though ED pathway, upper bound of PEP through PPP, lower bound of PYR from MAL, OAA from PEP, PEP from OAA; see also Zamboni et al., BMC Bioinformatics 2005, 6:209) and three external fluxes (glucose uptake rate, acetate excretion rate and growth rate) are available. All fluxes are part of a central carbon metabolism model for E. coli with stoichiometric matrix **S** (see Supplementary Table 2). In our formulation, we call R_s^i (*s = 1,…,11* ; *i=1,…,6*) the measured pathway branch ratio i of strain s and E_s^j (*s = 1, …, 11* ; *j=1,2,3*) the measured value of external flux *j* of strain *s*. Each flux ratio R_s^i can be expressed in terms of the flux vectors, appropriately weighted by two vectors $\boldsymbol{a_i} \in R^{\boldsymbol{n}}$ and $\boldsymbol{b_i} \in R^{\boldsymbol{n}}$:

$$\boldsymbol{a_i}'\mathbf{x}/\boldsymbol{b}_i'\mathbf{x} = R_s^i,\ i = 1, \ldots, 6, \qquad \text{[S11]}$$

These equations can be reformulated as standard linear equations:

$$\mathbf{Ax} = \mathbf{0}, \qquad \text{[S12]}$$

where the *ith* row of **A** is $\boldsymbol{a}_i' - \boldsymbol{b}_i' R_s^i$, *i = 1,…,6*. Translating the measured ratios and external fluxes to a feasible flux distribution for strain *s* is posed as the following optimization problem

$$\begin{aligned} \min \quad & \left|\left|\boldsymbol{A}\mathbf{x}\right|\right|^{\mathbf{2}} \\ s.t. \quad & \boldsymbol{S}\mathbf{x}_s = \mathbf{0}, \\ & E_s^j - \beta * std_s^j \le \mathrm{x}_s^j \le E_s^j + \beta * std_s^j,\ j = 1,2,3, \end{aligned} \qquad \text{[S13]}$$

where β is a coefficient determining the feasible range (here, we set $\beta = 1$), and std_s^j is the standard deviation of the measurements of external flux *j* of strain *s*.

The problem (S13) is a standard quadratic programming problem yielding the flux distribution, which is the closest one to the measured pathway branch ratio and is consistent with the stoichiometry constraints and external flux measurements. The problem can be solved efficiently and global optimality can be guaranteed. The optimal solution $\mathbf{x}_s$ of (S13) is the feasible flux distribution for strain *s* and can be used to test our invFBA algorithm.
