# Supplementary material for: Mapping the landscape of metabolic goals of a cell
Source: Genome Biol. 2016 May 23;17:109. doi: 10.1186/s13059-016-0968-2 (PMC4878026; doi:10.1186/s13059-016-0968-2)
Supplement: Additional file 11: Figure S1. — shows the results of a robustness analysis of OVA-predicted optimization of respiration in evolved E. coli strains. (PDF 144 kb) [file 13059_2016_968_MOESM11_ESM.pdf]

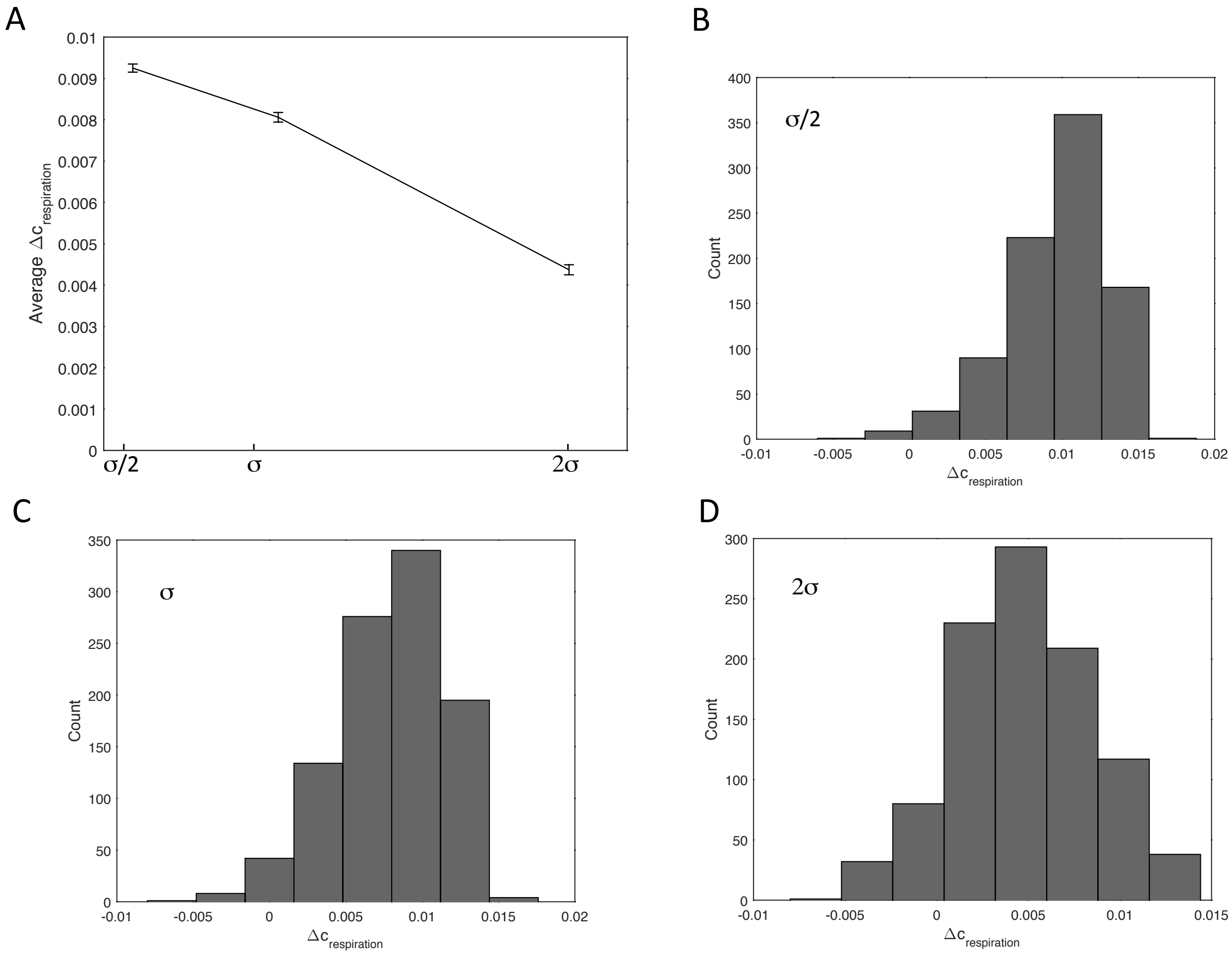

Figure S1 – Robustness of OVA-predicted optimization of respiration in evolved *E. coli*

This figure provides a sensitivity analysis associated with Fig. 5B in the main text, which portrays projections of the space of possible objective functions for evolved *E. coli* strains (with uptake/secretion fluxes shown in Fig. 5a). A key result of Fig. 5B is the gap ($\Delta c_{respiration}$) between the maximal component of the objective function along the "Respiration" axis (variable $c_{respiration}$) between two types of evolved strains (red and blue in Fig. 5B). For these calculations, absolute fluxes were inferred from measured flux ratios and exchange fluxes in evolved *E. coli* strains before serving as input to OVA. To test whether computational predictions were sensitive to experimental error, we modified these flux ratios and exchange fluxes by adding Gaussian noise. In particular, for each measured flux ratio and exchange flux (with mean value μ_i), a new noisy value was randomly sampled from a normal distribution with a mean μ_i, and a given standard deviation ($\sigma_i/2$, σ_i, $2\sigma_i$, where $\underline{\sigma_i}$ is the standard deviation associated with experimental measurements of quantity *i*). For each choice of noise Gaussian width, the random sampling process was repeated a thousand times, allowing the inference of a thousand absolute flux distributions. To estimate $\Delta c_{respiration}$ from each of these flux distributions, we applied OVA and computed the difference in the average maximal $c_{respiration}$ between high- and low-respiring strains (colored red and blue respectively in Figure 5a), at $c_{biomass}$=0 (the y-intercepts of Figure 5b). Figure S1a shows the mean $\Delta c_{respiration}$, at different degrees of noise ($\sigma_i/2$, σ_i, $2\sigma_i$), with error bars corresponding to standard deviations of the mean. One can see that as the noise level increases, the average $\Delta c_{respiration}$ gradually decreases to about half of its original value, but remains larger than zero, indicating robust greater optimality of respiratory activity of the "red" relative to "blue" strains. Figure S1b, c and d report the complete distributions of $\Delta c_{respiration}$ for the different levels of noise.
