# Supplementary material for: Mapping the landscape of metabolic goals of a cell
Source: Genome Biol. 2016 May 23;17:109. doi: 10.1186/s13059-016-0968-2 (PMC4878026; doi:10.1186/s13059-016-0968-2)
Supplement: Additional file 12: Figure S2. — illustrates the application of invFBA to two fully coupled reactions in long-term evolved E. coli strains. (PDF 105 kb) [file 13059_2016_968_MOESM12_ESM.pdf]

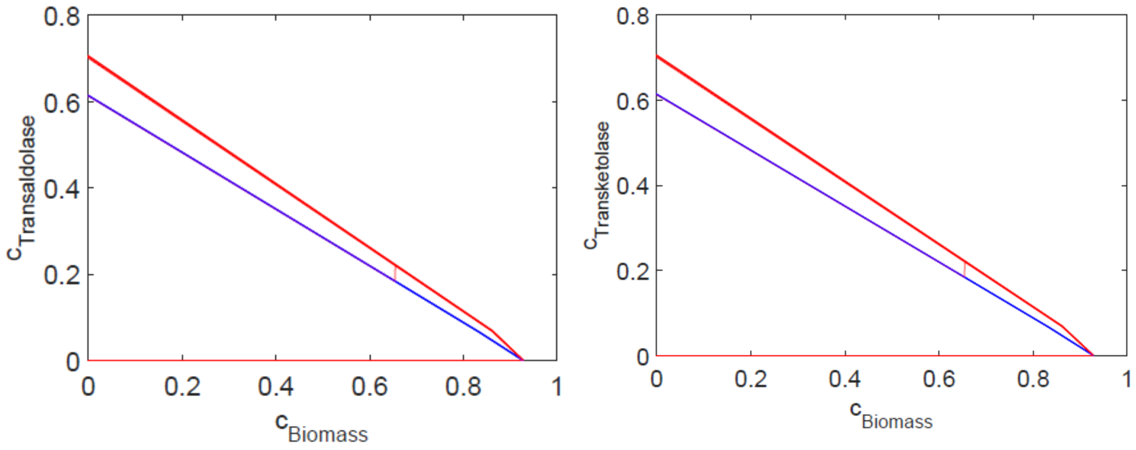

Figure S2 – Application of invFBA to two fully coupled reactions in long-term evolved *E. coli* strains

A projection (onto a two dimensional subspace) of the set of objective functions compatible with experimentally measured fluxes. The graph is obtained through a two-dimensional version of OVA: for each possible value of the growth flux coefficient of the objective function ($c_{Biomass}$), one can find the minimal and maximal value of the objective function coefficient for the transaldolase (Left, $c_{Transaldolase}$) or transketolase (Right, $c_{Transketolase}$) flux, obtaining areas that correspond to objective functions compatible with the measured fluxes. Such regions can be computed for the ancestral and all evolved *E. coli* strains. As shown in Table S5, the fluxes through these reactions are identical for each evolved *E. coli* strain, as one would expect from two fully coupled reactions.
