# Supplementary material for: Mapping the landscape of metabolic goals of a cell
Source: Genome Biol. 2016 May 23;17:109. doi: 10.1186/s13059-016-0968-2 (PMC4878026; doi:10.1186/s13059-016-0968-2)
Supplement: Additional file 13: Figure S3. — provides a schematic visualization of possible objective functions through invFBA, with L2-regularization. (PDF 182 kb) [file 13059_2016_968_MOESM13_ESM.pdf]

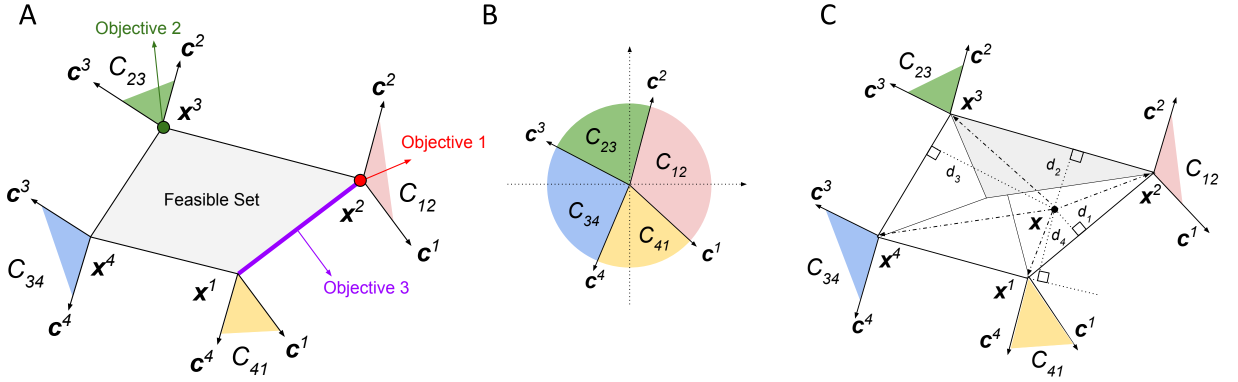

Figure S3 – Exploring possible objective functions through invFBA, with L2-regularization

(A) The feasible set of the FBA problem is a polyhedron – the intersection of points satisfying the linear constraints of Eq. 1. Extreme points of the feasible set are labeled as $\mathbf{x}^i$. Depending on the objective function coefficient vector c, one of these extreme points is the optimal solution found by FBA. In some cases, a particular c (*Objective 3*) can lead to multiple extreme points being optimal (purple edge). Note that for each extreme point x^i there are many different objective functions (i.e., different **c** vectors) that would give such point as an FBA optimum. For example, *Objective 1* would give x^2 as the optimum. However, any other objective between the two vectors $\mathbf{c}^1$ and $\mathbf{c}^2$ could lead to the same optimum. These objectives giving an equivalent FBA solution belong to a cone (C_{12}, in this case). The different cones of objective functions associated with the different extreme points are also depicted as shaded areas with different colors.

(B) The union of the cones of objective functions mentioned above is all of R^n ($n = 2$, in this case). If we use an L^2-norm regularization in the invFBA formulation (i.e., impose $\|\mathbf{c}\|=1$), valid **c** vectors lie on the surface of the unit ball in R^n.

(C) Illustration of the geometric intuition behind L^2-reguralized FBA. The FBA feasible polyhedron can be partitioned using bisector lines. In this two-dimensional example, the polyhedron is partitioned by 4 bisectors into 4 (colored) polytopes. For example, if the observed flux distribution **x** lies in the yellow polytope then c_1 is the corresponding objective function. If, however, the observed flux distribution lies in the bisector between the blue and the yellow region, invFBA will yield either $\mathbf{c}_1$ or $\mathbf{c}_4$.
